# Navigational expertise may compromise anterograde associative memory

**DOI:** 10.1016/j.neuropsychologia.2008.12.036

**Published:** 2009-03

**Authors:** Katherine Woollett, Eleanor A. Maguire

**Affiliations:** Wellcome Trust Centre for Neuroimaging, Institute of Neurology, University College London, 12 Queen Square, London WC1N 3BG, UK

**Keywords:** Hippocampus, Space, Navigation, MRI, VBM, Taxi drivers, Associative memory

## Abstract

Grey matter volume increases have been associated with expertise in a range of domains. Much less is known, however, about the broader cognitive advantages or costs associated with skills and their concomitant neuroanatomy. In this study we investigated a group of highly skilled navigators, licensed London taxi drivers. We replicated findings from previous studies by showing taxi drivers had greater grey matter volume in posterior hippocampus and less grey matter volume in anterior hippocampus compared to matched control subjects. We then employed an extensive battery of tests to investigate the neuropsychological consequences of being a skilled taxi driver. Their learning of and recognition memory for individual items was comparable with control subjects, as were working memory, retrograde memory, perceptual and executive functions. By contrast, taxi drivers were significantly more knowledgeable about London landmarks and their spatial relationships. However, they were significantly worse at forming and retaining new associations involving visual information. We consider possible reasons for this decreased performance including the reduced grey matter volume in the anterior hippocampus of taxi drivers, similarities with models of aging, and saturation of long-term potentiation which may reduce information-storage capacity.

## 1 Introduction

Grey matter volume increases in various parts of the brain have been identified in a number of skilled groups such as musicians (Gaser and Schlaug (2003); Munte, Altenmuller, & Jancke, 2002; Sluming et al., 2002; Sluming, Brooks, Howard, Downes, & Roberts, 2007), mathematicians (Aydin et al., 2007), bilinguals (Mechelli et al., 2004), jugglers (Draganski et al., 2004), and medical students (Draganski et al., 2006). As well as exploring the grey matter substrates of a skill itself, a related and important question is whether a skill and its associated neuroanatomy confer broader cognitive advantages or indeed costs. This question has particular significance for the domains of rehabilitation and education. Only a limited number of studies have considered the neuropsychological consequences of expertise. Professional musicians with increased grey matter volume in Broca's area were found to show enhanced judgements of line orientation and three-dimensional mental rotation ability (Sluming et al., 2002, 2007). This was attributed to their musical sight-reading and motor sequencing expertise. However, this expertise can come at a cost, with some musicians suffering focal dystonia, a loss of control and degradation of skilled hand movements (Munte et al., 2002).

The consequences of skill acquisition have also been investigated in another group of experts, London taxi drivers, who must know the layout of 25,000 streets as well as thousands of landmarks and places of interest around the city (Maguire et al., 2000; Maguire, Woollett, & Spiers, 2006a). The volume of the hippocampus in some non-human species has been reported to vary as a function of the demands placed on spatial memory (Barnea & Nottebohm, 1994; Biegler, McGregor, Krebs, & Healy, 2001; Lee, Miyasato, & Clayton, 1998; Smulders, Sasson, & DeVoogd, 1995; Volman, Grubb, & Schuett, 1997). Similar effects were also found in licensed London taxi drivers, with greater grey matter volume in posterior hippocampi and less grey matter volume in anterior hippocampi compared with control subjects. In addition, posterior hippocampal grey matter volume correlated positively, and anterior hippocampal grey matter volume negatively, with the number of years spent taxi driving (Maguire et al., 2000, 2006a). It has been suggested that the greater volume of posterior hippocampal grey matter in taxi drivers may be related to the acquisition, storage and use of the ‘mental map’ of a large complex environment (Maguire et al., 2000, 2006a). Related to this, Maguire et al. (2006a) confirmed that London taxi drivers performed significantly better than control subjects (London bus drivers) on tests assessing knowledge of London landmarks and their spatial relationships. Interestingly, in the same study taxi drivers were found to be significantly worse than control subjects on the delayed recall of the Rey–Osterreith complex figure (Osterrieth, 1944; Rey, 1941), a test of anterograde visuo-spatial memory. It was suggested that this below average score could be related to their reduced anterior hippocampal grey matter volume.

Unlike many other experts such as professional musicians and bilinguals, London taxi drivers acquire their knowledge in adulthood. In addition, taxi drivers are the only group where expertise has been consistently associated with both increases and decreases in grey matter volume. When considered along with the preliminary evidence of positive and negative test performances associated with their navigational expertise (Maguire et al., 2006a), this makes taxi drivers a particularly interesting model for studying the effects of expertise on the brain. Moreover, given the focal nature of the grey matter volume changes in the taxi drivers, they offer another line of evidence to complement neuropsychological and functional neuroimaging studies in helping to understand the role of hippocampus in memory and navigation.

Whilst Maguire et al.’s (2006a) preliminary neuropsychological findings in taxi drivers are intriguing, the battery of tests they employed was brief and did not permit a wide-ranging examination of cognitive and memory functions. In the current study, therefore, we sought to replicate and extend the previous findings by undertaking a comprehensive neuropsychological evaluation of a new sample of taxi drivers and matched control subjects. As well as assessing basic cognitive and affective functions, 22 different memory measures were taken. These tasks assessed a broad range of memory types and processes including visual and verbal memory, recall and recognition, single item and associative memory, and anterograde and retrograde memory. Our aim was to examine the memory profile of taxi drivers in the context of their navigational expertise, and to investigate whether any specific memory type or process was affected, positively or negatively. In so doing we hoped to contribute new information to debates about the functions of the human hippocampus specifically, and the benefits and costs of skill acquisition more generally.

## 2 Methods

### 2.1 Participants

Forty healthy male volunteers participated in the study. Of these, 20 were licensed London taxi drivers, and 20 were control subjects. All participants gave informed written consent to participation in the study in accordance with the local research ethics committee. The background details of the two groups are shown in Table 1. All taxi drivers had completed “The Knowledge” training, had passed the necessary Public Carriage Office examinations, and obtained a full (green badge) licence. For more on London taxi drivers and why London (UK) is particularly useful for testing navigation and related brain changes, see Spiers and Maguire (2007). None of the control subjects had worked as licensed London taxi drivers or mini-cab drivers. None was training to be a licensed taxi driver or had ever been involved in such training. The taxi drivers and control subjects did not differ in terms of age (*t*(38) = 1.81; *p* = 0.07), handedness (laterality index as measured using the Edinburgh Handedness Inventory, Oldfield, 1971) (*t*(38) = 0.90; *p* = 0.9), or age at which they left school (*t*(38) = 1.51; *p* = 0.1). As all participants were native English speakers, an estimate of verbal IQ was obtained using the Wechsler Test of Adult Reading (Wechsler, 2001). IQ estimates for both groups were in the average range, and did not differ significantly (*t*(38) = 0.57; *p* = 0.5). Visual information processing and abstract reasoning skills were assessed using the Matrix Reasoning sub-test of the Wechsler Abbreviated Scale of Intelligence (Wechsler, 1999). The mean scaled scores for both groups were comparable and did not differ significantly (*t*(38) = 0.36; *p* = 0.7).

### 2.2 MRI scan

Whole brain structural MRI scans were acquired on a 1.5 T Sonata whole body scanner (Siemens Medical Systems, Erlangen, Germany), with a whole-body coil for RF transmission and an 8-element phased-array head coil for signal reception, using a Modified Driven Equilibrium Fourier Transform (MDEFT) sequence (Ugurbil et al., 1993). Parameters were optimised as described in the literature (Deichmann, Schwarzbauer, & Turner, 2004; Howarth, Hutton, & Deichmann, 2005): for each volunteer, 176 sagittal partitions were acquired with an image matrix of 256 × 240 (Read × Phase). Twofold oversampling was performed in the read direction (head/foot direction) to prevent aliasing. The isotropic spatial resolution was 1 mm. Relevant imaging parameters were TR/TE/TI = 14.59 ms/3.4 ms/650 ms, BW = 96 Hz/Px, *α* = 20°. To increase the signal-to-noise ratio, an asymmetric position of the inversion pulse within the magnetisation preparation (duration TI) was chosen, and the delay between the initial saturation and the inversion amounted to 40% of TI (Deichmann et al., 2004). A fat saturation pulse was used to achieve fat suppression (see Howarth et al., 2005 for details). In addition, special RF excitation pulses were used to compensate for B1 inhomogeneities of the transmit coil (Deichmann, Good, & Turner, 2002). Images were reconstructed by performing a standard 3D Fourier Transform, followed by modulus calculation. No data filtering was applied either in *k* space or in the image domain. The total duration of the scan was 12 min.

### 2.3 Neuropsychological test battery

A test battery was employed to assess a range of cognitive, memory and affective functions, see Tables 2–4. The majority of tests were widely used standardised instruments and questionnaires with published normative data (see also, Maguire et al., 2006a; Maguire, Nannery, & Spiers, 2006b). A number of additional non-standard tests were also used and are detailed below.

#### 2.3.1 Public events test

Retrograde memory for public events was evaluated using 28 black and white photographs of well known public events spanning four decades (1974–2005). Participants were required to name the event, give the date of the event (±3 years) and name the location where the event took place. Only events where all 3 details were correct achieved a score of 1.

#### 2.3.2 London landmark recognition memory test

Recognition memory for London landmarks was assessed by showing subjects 48 colour photographs of landmarks one after another (see further details of this test in Maguire et al., 2006a, 2006b). Half of the pictures were of famous London landmarks and half were distractor landmarks that were neither famous nor in London, but were visually similar to the London landmarks. Target and distractor landmarks were randomly intermixed. The format was a yes/no recognition test where subjects were asked to state whether they recognised each landmark as a famous London landmark or not. The test was not formally timed, however subjects on average took 2–5 s per photograph.

#### 2.3.3 London landmark proximity judgements

Subjects’ knowledge of the spatial relationships between London landmarks was tested using a proximity judgements task. Stimuli were 10 colour photographs each depicting a famous London landmark. On each trial, subjects had to judge which of two other London landmarks was closer (as the crow flies) to the target London landmark (see Fig. 2 for an example stimulus). There were 10 trials. The test was not formally timed, however subjects on average took 5–8 s per photograph.

The key to the expertise in taxi drivers is of course their ability to navigate London's 25,000 streets. In this study we did not test directly this navigation ability for two reasons. Firstly, we knew that all the taxi drivers were highly qualified given they had passed the Public Carriage Office's stringent examinations. Secondly, the control participants simply would not have been able to perform such a difficult task, and this would have potentially led to chance/floor effects. Thus the two landmark tests described in Sections 2.3.2 and 2.3.3 above were used as general proxies for spatial expertise whilst at the same time control participants were able to score above chance on these tests and so meaningful comparisons could be made.

#### 2.3.4 Recognition memory tests for unfamiliar landscapes and buildings

Visual memory was further probed using Recognition Memory Tests for two categories of unfamiliar topographical stimuli, namely landscapes and buildings. The presentation and testing procedures for both the landscapes and buildings tests were identical to that of the standard Warrington Recognition Memory Test (Warrington, 1984; see also Cipolotti & Maguire, 2003). The target stimuli in each test were 50 black and white photographs of buildings, and 50 black and white photographs of landscapes. House numbers, people, animals and vehicles were all excluded from the photographs. The distractor items were chosen based on extensive pilot testing to be visually similar to the targets. As with the standard Recognition Memory Test, during the study phase the participant was instructed to decide if each landscape (or building) was pleasant or unpleasant. This was followed by a test phase in which the target and distractor items were randomly intermixed and presented in pairs. The subject was instructed to indicate which one of the items from each pair had been presented during the study phase.

#### 2.3.5 Object–place associations test

The ability to form and recall object–place associations was examined using a table top array (similar to those employed by Smith & Milner, 1981; and Crane & Milner, 2005). This comprised 16 coloured photographs of single objects placed on a white board measuring 64 cm × 48 cm (see Fig. 4). Subjects were given 60 s in which to name each object and study their positions. Immediately after, the array was removed and a blank board of equal dimensions was introduced. Subjects were given the 16 object photographs and asked to place them in the correct locations. Locations were noted by the examiner before the next learning trial. Subsequent study periods lasted 30 s and after each study period participants were immediately asked to reproduce the array as described above. No feedback was given during the test. The study-recall procedure was repeated until a criterion of 100% correct object placements was reached. Subjects were also asked to reproduce the array after a delay period of 30 min. The number of correct and incorrect positions was determined using a transparent template grid comprising squares of equal size (8 cm × 6 cm each). The exact position of correctly and incorrectly placed objects was recorded. A correct score of 1 was given to objects placed within 3 cm in any direction of their original place on the board. Two measures were derived, the number of learning trials required to achieve criterion, and the number of correct object–place associations recalled following the delay.

### 2.4 Procedure

Each subject was tested individually during two sessions each lasting approximately 2.5 h. The two sessions were at least 1 week apart and no more than 3 weeks apart. The order of neuropsychological tests within and across sessions was carefully balanced to ensure that similar tests were not administered in close proximity (for example, the Rey and Taylor complex figure tests were always administered in separate sessions). The order of neuropsychological testing and MRI scanning was random across subjects.

### 2.5 Data analysis

MRI images were analysed using voxel-based morphometry (VBM) implemented in the Statistical Parametric Mapping software (SPM5, Wellcome Trust Centre for Neuroimaging, London, UK). This method, which permits automatic whole-brain analysis, has been described in detail elsewhere (Ashburner & Friston, 2005; Maguire et al., 2006a; Mechelli, Price, Friston, & Ashburner, 2005). Briefly this involves a number of fully automated pre-processing steps including extraction of brain, spatial normalisation into stereotactic (MNI) space, segmentation into grey and white matter and CSF compartments, correction for volume changes induced by spatial normalisation (modulation), and smoothing with an 8 mm full-width at half-maximum isotropic Gaussian kernel. Analyses focussed on grey matter. The two groups (taxi drivers, control subjects) were compared using a two sample *t*-test to investigate differences in grey matter volume. In addition, parametric effects on grey matter volume of participant characteristics and behavioural performance were also examined. The effects of global grey matter volume and subject age were excluded by modelling them as confounding variables. Given that we used modulation, VBM in this context is comparing the absolute volume of grey matter structures. Our use of total grey matter volume as a covariate of no interest allows us to observe brain regions of absolute grey matter volume difference that cannot be explained by total grey matter volume differences. Given our apriori interest in the hippocampus, the significance level was set at *p* < 0.05 corrected for the volume of the hippocampus using a sphere of 4 mm. The significance level for the rest of the brain was set at *p* < 0.05 corrected for multiple comparisons across the whole brain. Our interest was in anterior and posterior hippocampal regions. As far as we are aware, there is no widely agreed system for formally classifying a grey matter volume difference as being located in the anterior or posterior hippocampus. In a previous structural MRI study we defined anterior, body and posterior hippocampus with respect to slices derived from a standard region of interest analysis (see p. 4399 of Maguire et al., 2000). In subsequent studies, including this one, we used these divisions to guide our labelling. In the current data set the anterior and posterior peaks are clearly distinct in the anterior–posterior (‘*y*’) direction and are separable by substantially more than the smoothing kernel.

For the behavioural data, basic group comparisons relating to participant characteristics were made using two-tailed *t*-tests. For the main analyses, data were screened for outliers, homogeneity of variance, and to ascertain if the data were normally distributed. Multivariate analysis of variance (MANOVA—Hotelling's trace multivariate test) was employed using the general linear model with the significance threshold set at *p* < 0.05. Three separate MANOVAs were performed where group (taxi drivers, control subjects) was the independent variable, and the basic cognitive measures/stress measures/memory measures were the dependent variables. Where a MANOVA indicated a significant effect, the between-subjects tests were employed to ascertain the source of the significance with a threshold of *p* < 0.05.

## 3 Results

### 3.1 MRI data

The main focus of this study was to investigate the neuropsychological profile of licensed London taxi drivers. However, we first sought to establish if the patterns of hippocampal grey matter volumes observed in previous studies of taxi drivers (Maguire et al., 2000, 2006a) were also true of the present sample. In particular, we were interested to know if they too would show a decrease in anterior hippocampal volume relative to control subjects, and a negative correlation between years taxi driving and grey matter volume in this region.

**Fig. 1:**
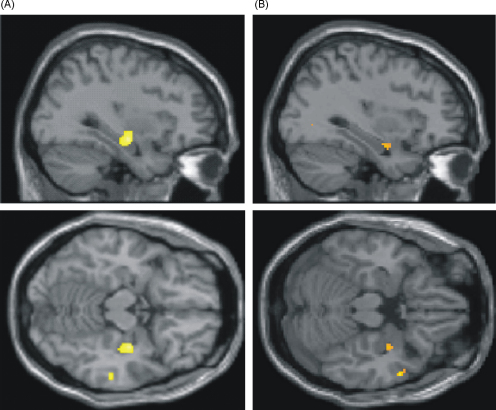
Anterior hippocampal grey matter volume differences between taxi drivers and control subjects. (A) Anterior hippocampal grey matter volume was decreased in licensed London taxi drivers compared with matched control subjects. Data are shown on sagittal (upper panel) and axial (lower panel) sections from the canonical SPM5 MRI scan. (B) Anterior hippocampal grey matter volume was negatively correlated with navigation experience, with less grey matter volume the more years spent taxi driving. Data are shown on sagittal (upper panel) and axial (lower panel) sections from the canonical SPM5 MRI scan.

In the first instance the two groups were compared to assess differences in grey matter volume. Greater grey matter volume was found in the control subjects compared to the taxi drivers in the right anterior hippocampus (peak (*x*, *y*, *z*) 34, −14, −14; *z* = 3.31; extent anteriorly in the *y* direction −9 mm extending to −22 mm posteriorly; see Fig. 1a). Taxi drivers had greater grey matter volume in the left posterior hippocampus (−24, −34, 6; *z* = 2.70) compared to control subjects. Taking into account the smoothing kernel (8 mm), these findings are comparable with those reported previously (Maguire et al., 2000, 2006a). No significant effects were apparent anywhere else in the brain.

We next examined the effect of navigation experience on grey matter volume by entering number of years taxi driving in London as a covariate of interest in the VBM analysis (controlling for subject age—see Section 2). As in the previous studies, grey matter volume in the right anterior hippocampus (34, −4, −20; *z* = 3.41) was found to decrease the longer taxi drivers had been navigating in London (see Fig. 1b). Right posterior hippocampal grey matter volume increased with number of years taxi driving (although this just failed to reach statistical significance, 12, −34, 2; *z* = 2.36; *p* = 0.06). No significant effects were apparent anywhere else in the brain.

### 3.2 Neuropsychological data

Having established the pattern of hippocampal grey matter volume was comparable with previous taxi drivers findings, we next turned to the main aim of the study, namely to establish if there were neuropsychological consequences of being a licensed London taxi driver. Mean performance scores are shown in Tables 2–4. Three separate MANOVAs were used to interrogate the data (see Section 2).

A MANOVA was first performed on the 8 basic cognitive measures listed in Table 2. There was no overall difference between the groups (*F*(8, 31) = 1.22; *p* = 0.32). This suggests that attention, working memory, spatial span, executive functions, construction abilities and perception are not variables that distinguish between the groups, and are presumably unlikely to explain the hippocampal grey matter volume differences that were observed. This was also the case for the 5 stress/anxiety measures listed in Table 3, as taxi drivers and control subjects did not differ significantly on this set of measures either (*F*(5, 34) = 0.89; *p* = 0.49).

**Fig. 2:**
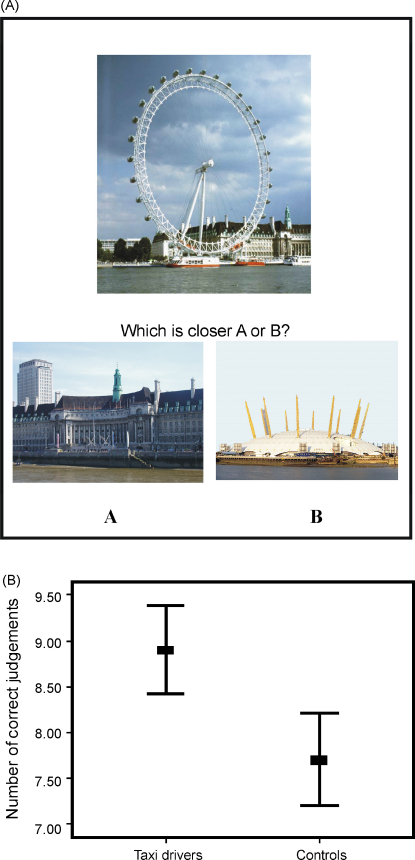
London landmark proximity judgements test. (A) An example stimulus with the target London landmark at the top. Participants had to decide which of the two lower landmarks (A or B) was closer, as the crow flies, to the target landmark. (B) Taxi drivers were significantly better than control subjects at making proximity judgements. Bars represent ±2 standard errors.

**Fig. 3:**
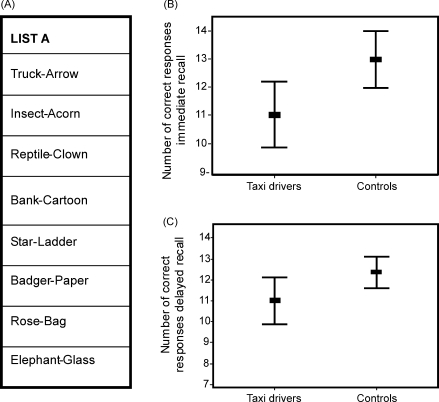
Verbal paired associates test. (A) The set of word pairs from the WMS-III verbal paired associates test. (B) Taxi drivers made significantly fewer correct responses during immediate recall and (C) delayed recall on this test compared with control subjects. Bars represent ±2 standard errors.

**Fig. 4:**
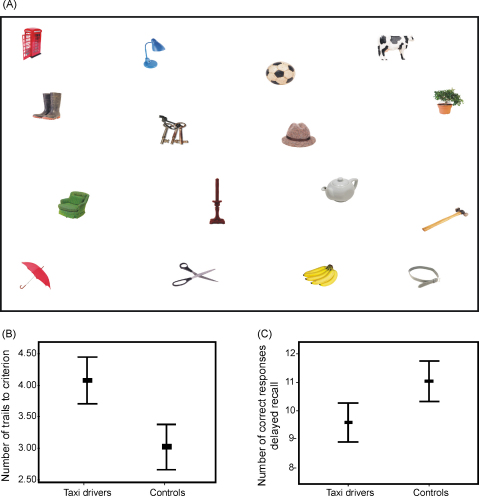
Object–place associations test. (A) The table top array used in the object–place associations test—see Section 2. (B) Taxi drivers required more learning trials to reach criterion than control subjects. (C) Taxi drivers recalled fewer of the object–place associations than control subjects following a delay. Bars represent ±2 standard errors.

A MANOVA was then performed on the memory measures listed in Table 4. On this occasion there was a significant difference between the two groups (*F*(22, 17) = 3.26; *p* = 0.01). The source of this difference was investigated using the tests of between-subjects effects produced by MANOVA. There were 8 main effects. Taxi drivers were significantly better than control subjects on the London landmark recognition memory test (*F*(1, 38) = 4.08; *p* = 0.05) and the London landmark proximity judgements test (*F*(1, 38) = 13.02; *p* = 0.001; see also Fig. 2). They also performed better than control subjects on the semantic section of the Autobiographical Memory Interview (AMI; *F*(1, 38) = 1.34; *p* = 0.01). By contrast, taxi drivers scored significantly worse than control subjects on the delayed recall of the Rey complex figure (*F*(1, 38) = 9.08; *p* = 0.005), the verbal-paired associates test (see also Fig. 3) both at immediate (*F*(1, 38) = 7.09; *p* = 0.01) and delayed recall (*F*(1, 38) = 4.51; *p* = 0.04). Taxi drivers also took significantly longer to reach criterion on the object–place association test (see also Fig. 4) (*F*(1, 38) = 18.25; *p* = 0.0001), and recalled fewer of the object–place associations after a delay (*F*(1, 38) = 9.36; *p* = 0.004).

### 3.3 Correlations between MRI and neuropsychological data

Taking the memory measures that distinguished between the two groups, we next examined whether performance on these tests correlated with grey matter volume. In taxi drivers, performance on the London landmark proximity judgements test correlated positively with grey matter volume in the posterior hippocampi (30, −30, 0, *z* = 3.59, *r* = 0.65, *p* < 0.002; −28, −30, 0; *z* = 2.87; *r* = 0.52, *p* < 0.02). In control subjects, performance on the delayed recall of the Rey complex figure correlated positively with mid-hippocampal grey matter volume (36, −24, −8, *z* = 2.90, *r* = 0.60, *p* < 0.005; −24, −32, 6, *z* = 2.98, *r* = 0.62, *p* < 0.003), as did immediate (−36, −22, −14, *z* = 3.57, *r* = 0.7, *p* < 0.0001) and delayed recall (30, −18, −20, *z* = 3.11, *r* = 0.57, *p* < 0.009) of the verbal paired associates test. Finally years experience taxi driving did not correlate significantly with any of the memory scores.

## 4 Discussion

In this study we found that licensed London taxi drivers, whilst being experts in their knowledge of London's layout, were deficient compared with matched control subjects at acquiring and retaining certain types of new information. Specifically, they were poorer at learning object–place and word-pair associations. After a delay they also recalled less of this associative information, and fewer elements of the Rey complex figure. By contrast their learning of and recognition memory for individual items was comparable with control subjects, as were retrograde memory for autobiographical and semantic information, executive and perceptual functions, working memory and levels of stress and anxiety.

That taxi drivers were significantly more knowledgeable about London landmarks and their spatial relations than the control subjects replicates a finding reported in a previous study (Maguire et al., 2006a). The same is true for their poorer delayed recall of the Rey complex figure (Maguire et al., 2006a). In the current study we were able to extend this line of enquiry further by employing a broader range of memory tests. We found that in addition to poorer performance on delayed recall of the Rey complex figure, the taxi drivers took significantly more trials to learn the locations of 16 objects on a table top array. Despite reaching criterion during the learning phase, they also recalled fewer of these object–place associations after a delay. The combination of these findings suggests that taxi drivers are poorer than control subjects at acquiring and retaining associations between objects (or lines in the case of the Rey complex figure) and locations. However, taxi drivers were also worse at associating pairs of words, both at immediate and delayed recall. This could suggest a broader associative deficit within visual and verbal domains. Whilst this may be the case, we note that the words used in the WMS-III verbal paired associates test are highly imageable (see Fig. 3). In our view it is likely that this test loads heavily on the visual domain and that overall the taxi drivers’ can be characterised as having poorer anterograde visual associative memory. Support for this comes from their performance on another verbal test that would seem to depend on associative processing. Their scores on the immediate and delayed recall of short stories (Logical Memory, WMS-III) were comparable with the control subjects. In addition, their learning over five trials of a list of words (RAVLT) was also similar to the control subjects.

Whilst we believe the neuropsychological findings point to a reduced facility for anterograde visual associative memory in taxi drivers, it is necessary to consider some additional issues. One of the subtests of the Doors and People Test requires subjects to associate the names and professions of four people (Baddeley, Emslie, & Nimmo-Smith, 1994). Taxi drivers and controls subjects did not differ on this task. This subtest, with just four associative items, whilst it may be sensitive to pathology, is not a difficult test for healthy individuals and is considerably easier than the other associative tests in our battery (such as the 16 item object–place associations test and the 18 item Rey Complex Figure test). We believe that the ease of this subtest meant that in this particular case it was not sensitive enough to differentiate between these two healthy age and IQ-matched groups.

In addition to the Rey complex figure, we also included the modified Taylor figure (Hubley & Tremblay, 2002; Taylor, 1979), to examine if poor performance extended to other complex figures. However, there was no difference between the groups on the delayed recall of the Taylor figure. Whilst it has been suggested that the two figures are comparable (Hubley and Jassal, 2006), Casey, Winner, Hurwitz, and DaSilva (1991) found that the Taylor figure lends itself to a verbal strategy to a greater degree than the Rey figure. That the taxi drivers were able to make associations within the verbal domain as noted above, may explain the lack of difference between the two groups on this test, and suggests that the Rey figure may be a purer measure of visuo-spatial memory than the Taylor figure.

Having established that taxi drivers show reduced performance on several anterograde visual associative memory measures, why might this be? It could be associated with the reduced grey matter volume in their anterior hippocampi. The anterior hippocampus in taxi drivers may be less efficient at forming associations. Even when this information is learned to criterion, there may be limited capacity for further consolidation and storage in the posterior hippocampus, given its involvement in supporting the complex spatial representation of London. The evidence for this, however, is not clear-cut. Performance of taxi drivers on the test of London landmark spatial relations correlated with right posterior hippocampal grey matter volume. However, their scores on the anterograde associative tasks on which they performed more poorly than the control participants did not correlate with grey matter volume. This echoes a finding from the previous study where taxi drivers were also poorer at the delayed recall of the Rey complex figure and their scores did not correlate with grey matter volume (Maguire et al., 2006a). Similarly, in this and the previous study the relationship between years experience taxi driving and performance on the memory tests also failed to reach statistical significance. Performance on the delayed recall of the Rey complex figure and the verbal paired associates test did, however, correlate with mid-hippocampal grey matter volume in control subjects in the current study. The peak voxels in several of these grey matter-memory correlations are within the spatial extent of the area showing decreased grey matter volume in taxi drivers relative to controls. However, the correlation peaks are generally more posterior. The reasons for the puzzling correlations are not clear, but are typical of the literature on this issue where Van Petten (2004) reports difficulties in finding consistent hippocampal volume correlates of standardised memory tests in healthy individuals in the age-range of our participants. Indeed, even in the case of patients, the literature reports only modest relationships between hippocampal volume and severity of memory deficit (Bigler, 2001; Di Paola et al., 2008).

In our case, the anterior hippocampal volume measures in taxi drivers did not have a limited range nor were they skewed. We speculate that the lack of correlation in taxi drivers might reflect an early and adverse impact of taxi training on anterograde visuo-spatial memory, and so a categorical difference rather than a linear relationship with grey matter volume results. Of note the variance was low on these tasks. The correlation data, however, highlight the difficulty of extracting a clear message from this aspect of our study. A longitudinal study of trainee taxi drivers (currently underway) may offer additional insights into the time course of anterior and posterior grey matter volume changes and their neuropsychological consequences.

What mechanisms might underpin the reduced associative memory processing observed here? In some ways it can be likened to a model of hippocampal processing in aging where it has been proposed that prior memories become the dominant pattern of the hippocampus to the detriment of the ability to encode new information (Wilson, Gallagher, Eichenbaum, & Tanila, 2006). Whilst the taxi drivers in the current study were relatively young, the extraordinary amount of information they have acquired, information that is known to be dependent on the hippocampus (Maguire et al., 2006b), may mimic the effects of an aged hippocampus, with concomitant changes in the balance of information processing. Alternatively, in vivo studies have shown that widespread synaptic strengthening on a population of neurons can cause a shut-down in long-term potentiation (LTP), and may reduce the information-storage capacity of the neuronal circuit (Roth-Alpermann, Morris, Korte, & Bonhoeffer, 2006). In the case of taxi drivers, experience-driven grey matter volume changes may push the neuronal circuit close to its limits and so alter the way the hippocampus processes new information.

The poorer learning ability in licensed London taxi drivers documented in this and the previous study (Maguire et al., 2006a), suggests these findings are robust. Whilst the results are intriguing, we acknowledge they are not easily explained and the theoretical implications that can be drawn are somewhat limited at this time. Future studies are required to explore the nature of associative memory in taxi drivers in more depth. This should include establishing if indeed the problem is definitively within the visual domain or whether it might be better characterised as difficulty with between-domain associations (Mayes, Montaldi, & Migo, 2007). It will be particularly important to devise tests that involve the same kind of spatial information on which the taxi drivers excel, namely large-scale spatial information that permits navigation. Such tests should examine whether or not taxi drivers can integrate new information into their established representation of London's layout, and also if they can learn the layout of an entirely new town or city.

In conclusion, the primary focus of this study was to undertake an in-depth neuropsychological assessment of licensed London taxi drivers which has not been reported hitherto. In so doing, our study shows that it is not enough to focus on the positive effects of being skilled, but that there may be costs associated with expertise that also need to be identified and considered.

## Funding source

This work was funded by the Wellcome Trust.

## Figures and Tables

**Table 1:** 

Participant characteristics	Taxi drivers mean (S.D.)	Controls mean (S.D.)
Age (years)	43 (3.46)	40 (6.54)
Education (age left school, years)	16.30 (0.80)	16.70 (0.86)
Estimated verbal IQ (WTAR)	98 (5.10)	98.90 (4.59)
Matrix reasoning scaled score (WASI)	11.75 (1.68)	12 (2.55)
Handedness–laterality index^a^	78 (45.91)	79 (19.32)
Years experience taxi driving	12.35 (6.85)	–

WTAR = Wechsler Test of Adult Reading; WASI = Wechsler Abbreviated Scale of Intelligence

a Edinburgh Handedness Inventory.

**Table 2:** 

Basic cognitive measures	Taxi drivers mean (S.D.)	Controls mean (S.D.)
Digit span scaled score (WAIS-III)	12.25 (2.57)	11.45 (2.01)
Spatial span scaled score (WMS-III)	12.90 (2.67)	12 (2.40)
Verbal fluency—FAS (total score)	43.60 (10.01)	47.40 (12.43)
Block design scaled score (WASI)	8.85 (1.81)	9.45 (2.43)
Brixton test (6–7 = average–high average)	6.60 (1.14)	7.20 (1.19)
VOSP object decision (/20)	17.85 (1.78)	16.85 (1.72)
VOSP cube analysis (/10)	9.70 (0.51)	9.60 (0.59)
VOSP number location (/10)	9.65 (1.13)	9.75 (0.55)

WAIS-III = Wechsler Adult Intelligence Scale; WMS-III = Wechsler Memory Scale; WASI = Wechsler Abbreviated Scale of Intelligence; VOSP = Visual Object and Space Perception Battery.

**Table 3:** 

Stress measures	Taxi drivers mean (S.D.)	Controls mean (S.D.)
Perceived Stress Scale	12.75 (7.30)	11.65 (7.13)
State-Trait Anxiety Inventory (State)	31.45 (9.13)	28.10 (5.49)
State-Trait Anxiety Inventory (Trait)	38.10 (10.33)	35.50 (11.98)
Life stress rating^a^	4.40 (1.75)	4.80 (2.41)
Job stress rating^a^	5 (1.91)	4.50 (2.03)

a Ratings from 1 (no stress) to 10 (very high stress).

**Table 4:** 

Memory measures	Taxi drivers mean (S.D.)	Controls mean (S.D.)
Doors and People Test (overall scaled score)	9.70 (3.26)	9.95 (2.81)
WRMT: Faces scaled score	10.30 (3.11)	10.80 (3.25)
WRMT: Words scaled score	12.10 (1.83)	12.85 (1.22)
Autobiographical Memory Interview (semantic)^a^	53.10 (1.52)	49.02 (6.55)
Autobiographical Memory Interview (autobiographical)	22.25 (3.29)	23.20 (1.60)
Public events test (/28)	20.05 (10.49)	17.00 (5.08)
Rey auditory verbal learning test: IR (Σ for 5 trials/75)	54.90 (6.92)	57.10 (5.32)
Rey auditory verbal learning test: DR (/15)	10.55 (2.56)	12.00 (2.10)
Logical memory: IR (WMS-III) percentile	69.25 (19.47)	70.05 (22.67)
Logical memory: DR (WMS-III) percentile	68.70 (14.91)	69.05 (20.95)
Verbal paired associates: IR (WMS-III) scaled score^b^	10.85 (2.47)	12.80 (2.14)
Verbal paired associates: DR (WMS-III) scaled score^b^	10.75 (2.38)	12.10 (1.55)
London landmark recognition memory test (/48)^a^	41.10 (3.71)	37.60 (6.40)
London landmark proximity judgments (/10)^a^	8.90 (1.02)	7.70 (1.08)
Rey–Osterreith complex figure copy (/36)	35.65 (0.98)	36 (0)
Rey–Osterreith complex figure–DR (/36)^b^	19.72 (5.07)	24.85 (5.65)
Modified Taylor complex figure copy (/36)	35.75(0.91)	35.90 (0.44)
Modified Taylor complex figure–DR (/36)	19.35 (6.02)	22.35 (5.25)
Recognition Memory Test for unfamiliar buildings (/50)	41.60 (5.28)	43.85 (4.83)
Recognition Memory Test for unfamiliar buildings (/50)	44.05 (3.01)	45.75 (2.80)
Object–place associations: Number of trials to criterion^b^	4 (0.79)	2.95 (0.75)
Object–place associations: DR (number correct/16)^b^	9.40 (1.46)	10.85 (1.53)

WRMT = Warrington Recognition Memory Test; IR = immediate recall; DR = delayed recall after 30 min); WMS-III = Wechsler Memory Scale; The Autobiographical Memory Interview is the test devised by Kopelman, Wilson, and Baddeley (1990).

a Taxi drivers significantly better than control subjects.

b Control subjects significantly better than taxi drivers.
